# Autochthonous Case of Eosinophilic Meningitis Caused by *Angiostrongylus cantonensis*, France, 2016

**DOI:** 10.3201/eid2306.161999

**Published:** 2017-06

**Authors:** Yann Nguyen, Benjamin Rossi, Nicolas Argy, Catherine Baker, Beatrice Nickel, Hanspeter Marti, Virginie Zarrouk, Sandrine Houzé, Bruno Fantin, Agnès Lefort

**Affiliations:** Hôpital Beaujon, Clichy, France (Y. Nguyen, B. Rossi, C. Baker, V. Zarrouk, B. Fantin, A. Lefort);; Hôpital Bichat, Paris, France (N. Argy, S. Houzé);; Swiss Tropical and Public Health Institute, Basel, Switzerland (B. Nickel, H. Marti);; University of Basel, Basel (B. Nickel, H. Marti)

**Keywords:** eosinophilia, meningitis/encephalitis, *Angiostrongylus cantonensis*, helminths, parasites, France

## Abstract

We report a case of a 54-year-old Moroccan woman living in France diagnosed with eosinophilic meningitis caused by *Angiostrongylus cantonensis.* Diagnosis was based on clinical symptoms and confirmed by testing of serum and cerebrospinal fluid samples. Physicians should consider the risk for *A. cantonensis* infection outside of endemic areas.

*Angiostrongylus cantonensis* is a rat lungworm that has long been recognized as a cause of eosinophilic meningitis in Southeast Asia, the Pacific Islands, and the Caribbean, where it is endemic (*1*)*.* Although sporadic imported cases have been described in European travelers (*2**–**5*)*,* no autochthonous case of eosinophilic meningitis caused by *A. cantonensis* worms has been reported previously in metropolitan France.

A 54-year-old Moroccan woman was admitted to an emergency ward in Paris in 2016 because of fever and headache lasting 2 weeks. She had a history of type 2 diabetes treated with metformin and did not receive any other medication before the onset of symptoms. She was a pesco-vegetarian and worked as a cleaning woman in an office in Paris. She had taken up residence near Paris in the 1980s and had not traveled out of France since, except for Morocco over 2 years ago.

At admission, her blood pressure was 126/68 mm Hg, and her pulse was regular at 79 beats/min. The physical examination revealed meningeal signs with neck stiffness and photophobia without neurologic localization signs. No other clinical abnormality could be evidenced. Laboratory testing of serum showed a leukocyte count of 12.1 × 10^9^ cells/L (reference range 4.5–11.0 × 10^9^/L) with 18% (2.2 × 10^9^ cells/L) eosinophils and a C-reactive protein of 73 mg/dL (reference range 0.08–3.1 mg/L). Cerebrospinal fluid (CSF) analysis showed a leukocyte count of 950 cells/µL with 56% eosinophils, a glucose concentration within reference range at 0.4 g/L, and an elevated protein level of 0.9 g/L. CSF pressure was 16 cm H_2_O (0.16 kPa). CSF PCR tests for viral diseases (including Epstein-Barr virus and herpes simplex viruses 1 and 2); Gram stains and cultures for bacteria, mycobacteria, and fungi; and a search for malignant cells were all negative. Serologic tests specific for HIV and human T-lymphotrophic viruses 1 and 2 were negative. Parasitologic examinations of stool samples did not reveal any ova, cysts, or Charcot-Leyden crystals. Serologic tests of peripheral blood samples for *Toxocara* spp., *Trichinella* spp., *Schistosoma* spp., *Taenia solium* cysticercosis, *Gnathostoma* spp., and *Fasciola hepatica*, as well as serologic tests of CSF samples for *Gnathostoma*, were all negative. In contrast, antibodies directed toward the *A. cantonensis* 31-kDa antigen were detected by Western blot both in serum and CSF samples. Computed tomography (CT) and magnetic resonance imaging of the brain showed normal findings. Thoracic and abdominal CT showed a liver hypodensity without signs of malignancy. The patient was treated by evacuation of the CSF, prednisone (1 mg/kg equivalents for 1 wk), and albendazole (800 mg/d for 5 d), which lead to the complete regression of symptoms.

Eosinophilic meningitis is defined as a CSF eosinophil count above 10% of the total cell count, or exceeding 10 eosinophils/µL. The main etiologies of eosinophilic meningitis are parasitic infectious diseases (among which *A. cantonensis* and *Gnathostoma* spp. infections are the most frequent), hematologic or neoplastic disorders, adverse drug reactions, and primary eosinophilic meningitis (*6*). In this case, diagnosis of angiostrongyliasis was confirmed by Western blot. According to the literature, detection of the 31-kDa band by immunoblot shows a high sensitivity and specificity (>99% for both) for the diagnosis of *A. cantonensis* infections (*7*)*.*

Human infection by *A. cantonensis* lungworm results from the ingestion of uncooked paratenic hosts (freshwater shrimps, crabs, and frogs); intermediate hosts (snails and slugs); or poorly cleaned contaminated vegetables that have been in contact with these hosts (*8*)*.* After passage through the gastrointestinal tract, the larvae cross into systemic circulation and migrate to the central nervous system. The incubation period of this disease varies from 7 to 35 days (*1*)*.* Although angiostrongyliasis is mainly a tropical disease, this infection has become an emerging infection in Southeast Asia, the Pacific Islands, and the Caribbean Islands and in travelers who return home from endemic areas (*9*). Some authors have suggested that global warming, changes in dietary habits, increased global transportation of food products, increased international trade, and traveling could explain this expansion (*10*).

Because the patient we describe had not left the Paris area for 2 years, an infection by consumption of contaminated food in Morocco can be excluded; *A. cantonensis* larvae do not survive longer than a few weeks to a few months in the human body (*8*). She might have acquired the infection in France by consuming imported contaminated food, but she did not declare any consumption of imported snails or freshwater shrimp. She only declared eating vegetables and fish bought in a local supermarket. We failed to identify the source of infection or risky behaviors. Because of global food trade, physicians should be aware of the risk for *A. cantonensis* infection even in patients who do not report recent travel to endemic areas.
